# Dichotomy, Trichotomy, or a Spectrum: Time to Reconsider Attentional Guidance Terminology

**DOI:** 10.3389/fpsyg.2020.02243

**Published:** 2020-09-30

**Authors:** Hanna Benoni, Itay Ressler

**Affiliations:** Department of Psychology, The College of Management, Academic Studies, Rishon LeZion, Israel

**Keywords:** bottom-up control of attention, top-down control of attention, stimulus-driven, attentional guidance, saliency, visual attention

## Introduction

The distinct categories of ‘‘bottom-up'' and ‘‘top-down'' control of attention have been central to the majority of theories of visual attention for several decades (e.g., Jonides, 1981; Posner and Petersen, 1990; Wolfe, 1994; Kim and Cave, 1999; Itti and Koch, 2000). Given the widespread use of these terms, it is interesting that a closer look at the literature reveals that researchers differ in the ways they delineate the boundaries between the classes of attentional control. A recent discussion by Egeth (2018) and Gaspelin and Luck (2018), commenting on Theeuwes (2018), illustrates particularly well that researchers may interpret the concept of “top-down” control of attention differently.

In this opinion piece, we briefly describe and update the different taxonomies of attentional control. We elaborate on a recent view by Benoni (2018) and propose an alternative model, a “relevance spectrum,” to represent the various sources of attentional deployment. We end with a discussion on the problem of unstandardized terminology and a call to reconsider the necessity of the terms “top-down” and “bottom-up” in the lexicon of attentional guidance.

## The Different Taxonomies Dividing the Field of Attentional Guidance

Traditional approaches dichotomously classify “bottom-up” and “top-down” guidance of attention according to the *intention* and *volition* criteria. While “top-down” deployment of attention is described as a *voluntary* process driven by one's goals, “bottom-up” control of attention is described as an *involuntary* process driven by the physical saliency of the stimuli (e.g., Baluch and Itti, 2011; Pinto et al., 2013).

The classical dichotomy has been challenged by Awh et al. (2012) who argue that this taxonomy does not explain a great many phenomena and should therefore be changed to a trichotomy. For instance, the stimuli linked with high reward draw more attention than equivalently salient stimuli linked with low reward, including whenever this action counters current goals (Hickey et al., 2010). Awh et al. (2012) suggested an intermediate *third* category labeled “selection history” to represent all cases in which attentional allocation is neither consistent with current goals of the viewer nor driven by the physical features of the salient stimuli *per se* or generally, to all cases of lingering biases of previous selection (Bucker and Theeuwes, 2014; Stankevich and Geng, 2014; Munneke et al., 2015).

In a more recent article, Theeuwes (2018) presented an updated view where the aim was to further define the sources of attentional control. Theeuwes emphasized that the “top-down” category of attentional control is separate from all other instances based on the *volition* criterion. In the trichotomy, *voluntary* allocation of attention is a necessary condition for defining the process as “top-down” guidance. Both categories of “selection history” and “bottom-up” guidance are characterized by *involuntary* guidance. However, while “bottom-up” effects are exclusively driven by salience, “selection history” effects occur based on the past selection criterion (i.e., previous relevance).

Following Theeuwes's article (2018), Egeth (2018), and Gaspelin and Luck (2018), argued that there is no consensus on Theeuwes's characterization of attentional guidance. They claimed that instances of “selection history” are in fact examples of “top-down” guidance and contended against the intention criterion. Egeth (2018), Gaspelin and Luck (2018), and many others (e.g., Wolfe et al., 2003; Mysore and Knudsen, 2013) uphold the view that “top-down” is a broad category that consists of involuntary attentional deployment, not just voluntary, and may be driven by implicit goals, not just by explicit goals. Hence, their view is in favor of the dichotomy, but in a manner that would divide the field based on a *relevance* criterion, rather than *intention*. In this taxonomy, “top-down” guidance is driven by factors that are *relevant* to the organism's behavior, explicitly or implicitly, while “bottom-up” control is driven solely by physical properties.

**Figure 1:**
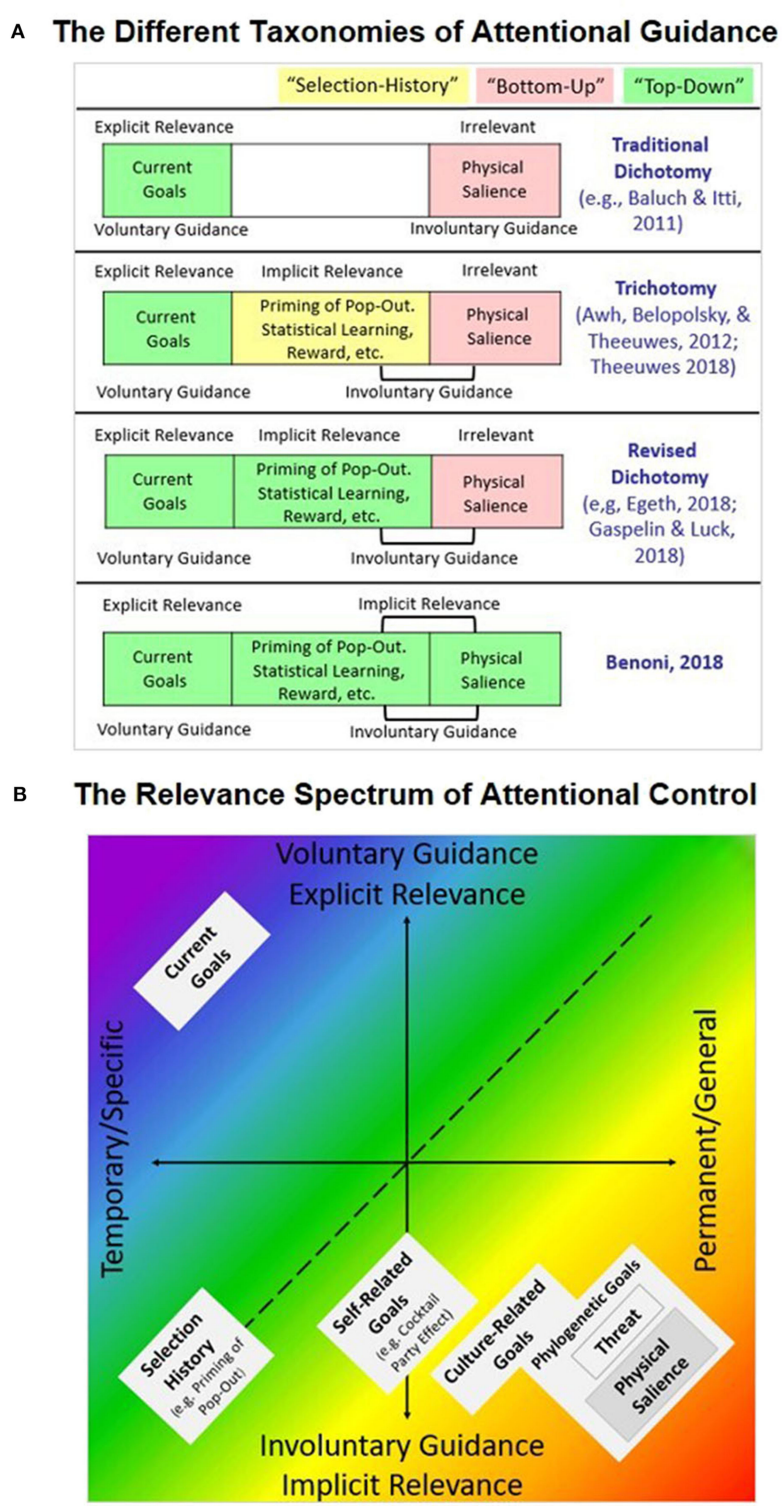
**(A)** An illustration of the different taxonomies dividing the field of attentional guidance. **(B)** A proposed abstraction for the relevance spectrum of attentional control. Each instance of attentional guidance is classified by two scales: volition scale: from voluntary attentional allocation guided by explicit relevance, to involuntary attentional allocation guided by implicit relevance. Temporality scale–from temporary and specific relevance to permanent and general relevance. Current goals that are characterized by voluntary guidance, guided by explicit and temporary relevance, signify one edge of this 2D space (up-left). Physical salience that may be characterized by involuntary guidance driven by implicit and hard-wired permanent relevance would signify the other edge of this spectrum space (down-right). As we move diagonally from up-left to down-right, the instances represent forms of relevance that are less consensually agreed upon as top-down instances by different researchers. All phenomena that reflect attentional bias toward specific stimuli in the visual field are represented here in this relevance 2D spectrum. This includes biases to prior selections [e.g., Awh et al. (2012)] that may by characterized by involuntary attentional allocation guided by implicit and temporary relevance. This also includes biases to stimuli representing permanent self-related goals [e.g., cocktail party effect; Moray (1959)] that may be characterized by involuntary guidance and fall halfway in between the temporary to permanent range, biases toward stimuli representing culture-related goals [e.g., Brosch and Sharma (2005)], and biases to stimuli representing implicit goals to perceive potentially life-threatening stimuli [e.g., Yorzinski et al. (2014)].

Finally, a recent study by Benoni (2018) questioned the *relevance* criterion that separates the “bottom-up” category from all other instances. The study demonstrated attentional allocation to *task-irrelevant* salient stimuli *prior* to the presentation of any external physical features. Hence, it demonstrated that the so-called stimulus driven effects may be initialized by a process that is internal to the observer ^1^. Benoni argued that the attentional system treats salient stimuli as *essentially relevant* and therefore continuously and implicitly seeks out salient items in the visual field. Thus, if implicit goals can be considered types of “top-down” control, attentional allocation to salient stimuli should also be considered a type of implicit “top-down” guidance (Figure 1 illustrates the different taxonomies).

## An Alternative View: The “Relevance Spectrum”

The study by Benoni (2018) proposed that all instances of attentional control may be represented by a goal-directed “top-down” variable (implicitly or explicitly). In this section, we seek to elaborate on that idea, complement that suggestion based on theoretical premises, and propose a “relevance spectrum” that may represent all instances of attentional allocation, and may replace the “top-down''–‘‘bottom-up” categorical variable of the sources of attentional control.

### Theoretical Tenets

Consonant with many other researchers' views (e.g., Wolfe et al., 2003; Mysore and Knudsen, 2013; Egeth, 2018; Gaspelin and Luck, 2018), the starting point of our view is the notion that top-down control of attention is a broad category that refers to every attentional process that is driven by factors relevant to the organism's behavior, explicitly or implicitly. However, contrary to all approaches, we argue that definition logically leads to the theoretical conclusion that “bottom-up” is just one of the top-down attentional guidance possibilities.

This claim requires an explanation; the common approach treats instances of “bottom-up” control of attention as processes that do not necessarily serve the organism's goals. The literature relies on the assumption that salient stimuli that differ from the observer's current goals are identified as irrelevant and suggest that attentional shifts toward such stimuli reflect the limitations of the attentional system. However, it has been asserted that salient items in the visual field are more informative and that attention is directed to informative locations (e.g., Itti, 2007; Bruce and Tsotsos, 2009; Benoni, 2018).

Indeed, we are built to attend to salient items, and it is reasonable to think that we are made this way with good reason. For instance, in a hypothetical deployment of attention that does not prioritize uniqueness and is not driven by goals, a singleton is statistically more likely to be missed (one vs. many) than identical non-singletons that provide repetitious semantic knowledge. This is because information received from repeated items is “more of the same”; it is enough for the observer to grasp only one of the non-singleton items in order to be aware of the information they are facing. Hence, to avoid semantic information loss and to maximize information gain, it would be ecologically efficient if the attentional system was tuned to prioritize the information that is prone to be missed (singletons). Thus, salient items may be relevant based on *phylogenetically implicit goals* to perceive ecologically significant visual items, which make them *essentially relevant*, though they are not task-relevant [see also Benoni (2018)].

Although the literature seems to overlook assertions that may lead to the conclusion that salient items may be identified as fundamentally relevant, it is likely that most theoreticians and researchers would accept the idea that there is a good reason for the system to attune to salient items. Accepting this idea means accepting the notion that salient items may be relevant based on implicit goals and reflect implicit goal-driven information. Additionally, as described above, many researchers accept the idea that the top-down category is broad and includes all cases in which attention is allocated to stimuli that may be relevant to the organism's behavior and reflect implicit goals (e.g., Wolfe et al., 2003; Mysore and Knudsen, 2013; Egeth, 2018; Gaspelin and Luck, 2018). Thus, those assertions coupled together make the consensus regarding the notion of a distinct bottom-up guidance of attention seem rather dubious. Those premises combined suggest that bottom-up attentional guidance may not be separate from top-down guidance and could be considered one of many top-down search possibilities.

### A Proposed “Relevance Spectrum”

Developing the aforementioned ideas, we propose that all instances of attentional guidance can be classified on a 2D spectrum space produced by two independent variables. (a) Volition scale: from voluntary attentional allocation guided by explicit relevance to involuntary attentional allocation guided by implicit relevance. (b) Temporality scale: from temporary and specific relevance to permanent and general relevance (see Figure 1 for illustration and explanations). Thus, current goals that are characterized by voluntary guidance guided by explicit and temporary relevance signify one edge of this space. Physical salience that may be characterized by involuntary guidance driven by implicit and permanent relevance would signify the other edge of this spectrum space. All phenomena that reflect attentional biases for specific stimuli in the visual field would be represented by the relevant space between those edges.

### Advantages

The “relevance spectrum” representation may hold certain advantages. The main advantages as we see them: (a) Even in the extended categorical representation, the trichotomy, some effects remain unclassified. For example, findings suggest that emotional stimuli summon attention (e.g., Anderson and Phelps, 2001; Flykt, 2005; Yorzinski et al., 2014). This type of attentional guidance may not be easily delineated in terms of low-level physical stimulus features [see Awh et al. (2012)]. Nor is it easy to classify such effects in the “selection history” category. On a spectrum, however, every instance could easily be represented. (b) The relevance spectrum view highlights the importance of salient items; instead of considering attentional allocations to task “irrelevant” singletons as limitations, such processes would be acknowledged as essentially effective processes. Hence, this view seems to be more consistent with the definition of attention or with the basic tenets of attentional research, which define attention as the mechanism in charge of resolving the problem of limited capacity, by ensuring that only relevant information is granted access to further processing (e.g., Broadbent, 1958; Tsotsos, 1990). This definition should apply to all types of attentional deployment.

## Unstandardized Terminology: The Problem and the Required Solution

Data, contents, and meanings are not contingent upon their labels. However, this does not mean that the problem of unstandardized terminology should be disregarded. The commentary by Gaspelin and Luck (2018) pointed out this problem, “If we cannot agree upon the meaning of “top-down” and “bottom-up,” there is little hope for reaching consensus about the mechanism of attentional control” (p. 1).

As researchers in the 21st century, we are overwhelmed with extensive research and information, available at the touch of a button. A tendency to only read titles, abstracts, and discussions, even skip paragraphs, in the process of familiarizing oneself with research literature, exacerbates the problem. If the phrase “top-down” means different things to different readers, the meanings and inferences can easily be misunderstood.

Egeth (2018) points out that “there is not necessarily a right or wrong answer to the question of how a field should be divided up” (p. 2). This assertion may imply that reaching a consensus on the definitions seems improbable. Therefore, in the meantime, we should at least consider a simple and immediate solution: *giving up* the use of the concepts “top-down” and “bottom-up” in the study of attentional guidance. Our suggestion that bottom-up guidance could be considered one of many top-down search possibilities provides additional justification to abandon the use of these terms. If all instances are essentially types of top-down effects, then the term “bottom-up” is inadequate, the term “top-down” is uninformative, and both terms may be regarded as unnecessary.

Instead of those definitions, we could use uncontroversial terms that stem from the characteristics of attentional guidance; for example, “involuntary attentional control” vs. “voluntary attentional control” (Figure 1A illustrates the consensus regarding these terms). Researchers' positions may differ as to whether a certain involuntary instance should be classified as a “top-down” or “bottom-up” process. By contrast, it is highly unlikely that they would disagree as to whether a certain process is “involuntary” or “voluntary.” In analogy to this, people may question the fact that a door is a piece of furniture, but it is unlikely that they would question the notion that a certain door is indeed a door.

## Author Contributions

HB conceived the idea presented in this opinion piece and wrote the majority of the manuscript. IR wrote sections of the manuscript. All authors contributed to the manuscript improvements.

### Conflict of Interest

The authors declare that the research was conducted in the absence of any commercial or financial relationships that could be construed as a potential conflict of interest.

## Footnotes

^1^See Benoni (2018) for differences between this view and the Contingent Capture account by Folk et al. (1992).
